# Case Report: Primary cutaneous infection caused by *Microascus trigonosporus*

**DOI:** 10.3389/fmed.2026.1963973

**Published:** 2026-09-24

**Authors:** Chunli Yang, Zheng Lin, Dongping Yang, Xiguang Xu

**Affiliations:** 1 Department of Dermatology, Affiliated Hospital of Jining Medical University, Jining, China; 2 Department of Emergency, Affiliated Hospital of Jining Medical University, Jining, China

**Keywords:** cutaneous infection, itraconazole, metagenomic next-generation sequencing (mNGS), *Microascus trigonosporus*, non-transplant host

## Abstract

**Background:**

*Microascus trigonosporus* is an emerging opportunistic filamentous fungus, with most reported cases involving invasive pulmonary infections in solid organ transplant recipients. Worldwide, cutaneous infections caused by this organism are extremely rare. This case highlights an unusual presentation of primary cutaneous infection in a non-transplant host.

**Methods:**

We report a case of a 67-year-old woman who presented with progressive skin erythema and pustules for two months, unresponsive to topical halometasone. Her medical history included glaucoma, hypertension, and coronary heart disease. The diagnostic workup included microscopic examination of skin lesion scrapings, skin biopsy for histopathology, fungal culture, and metagenomic next-generation sequencing (mNGS) for pathogen identification. The patient was treated sequentially with oral fluconazole for 2 weeks, followed by itraconazole for 8 weeks, and was followed up for 1 year.

**Results:**

Microscopic examination of skin scrapings revealed abundant, branched, and septate fungal hyphae. Skin biopsy revealed chronic inflammation with neutrophil infiltration in the superficial dermis. Fungal culture results were positive, and the isolate exhibited morphological features consistent with the genus *Microascus*. The pathogen was definitively identified as *M. trigonosporus* using mNGS. The skin lesions completely resolved after sequential antifungal therapy (fluconazole for 2 weeks, followed by itraconazole for 8 weeks). No drug-related adverse events were observed, and no recurrence was noted during the 1-year follow-up.

**Conclusion:**

This case expands the disease spectrum of *M. trigonosporus*, demonstrating that primary cutaneous infections can occur in non-transplant hosts. mNGS facilitates the rapid identification of rare filamentous fungi in chronic, undiagnosed skin lesions, enabling earlier and more precise diagnoses.

## 1 Introduction

The genus *Microascus* comprises environmental filamentous fungi that are increasingly recognized as emerging opportunistic pathogens in immunocompromised hosts (1). Among them, *Microascus trigonosporus* has attracted growing clinical attention, with most reported cases involving invasive pneumonia in solid organ transplant recipients, which is associated with poor prognosis (2). To date, cutaneous infections caused by *M. trigonosporus* have rarely been reported.

The genus *Microascus* is phylogenetically closely related to *Scopulariopsis*, and members of this group generally exhibit intrinsic resistance to fluconazole, which greatly complicates empirical antifungal therapy (3, 4). Chronic, atypical skin lesions caused by this pathogen lack specific features and are easily misdiagnosed as inflammatory or immune-mediated dermatoses. Inappropriate application of topical glucocorticoids may suppress local cutaneous immunity, potentially leading to disease exacerbation. Conventional fungal diagnosis relies on culture and morphological identification, which are time-consuming and heavily dependent on the expertise of laboratory personnel (4). Metagenomic next-generation sequencing (mNGS) enables the rapid detection of rare fungal pathogens directly from clinical specimens, thereby overcoming the limitations of conventional diagnostic methods (5–7).

We report a case of primary cutaneous infection caused by *M. trigonosporus* in a non-transplant patient. We summarize the clinical characteristics, diagnostic workup, treatment, and follow-up of this case to raise awareness of *M. trigonosporus* as a cause of cutaneous infection and to help clinicians avoid inappropriate empirical antifungal therapy.

## 2 Case description

A 67-year-old female farmer from rural Shandong Province presented to our dermatology clinic on 28 May 2025, with a 2-month history of pruritic erythema and pustules on the trunk and neck. The skin lesions initially appeared on the abdomen 2 months ago without an identifiable trigger. Topical halometasone cream (0.05%), which was initiated at another hospital and applied as a thin layer twice daily for 2 weeks, provided no improvement. The lesions progressively enlarged: some coalesced into confluent patches, others assumed an annular configuration, and new pustules continued to appear. Subsequent treatment with traditional Chinese medicine (details unknown) was also ineffective. The patient denied experiencing cough, sputum production, abdominal pain, diarrhea, or other constitutional symptoms. Her appetite and bowel habits remained normal, but her sleep was disturbed.

Her medical history included over 10 years of hypertension and coronary heart disease, as well as 2 years of glaucoma, for which she used long-term topical brinzolamide 1% eye drops. A family history of glaucoma was noted. There was no history of surgery, trauma, or blood transfusion, and the patient reported a history of penicillin allergy. The patient was a housewife with no recent travel history or animal contact and had not received immunosuppressive therapy prior to the onset of the disease.

Physical examination revealed stable vital signs: temperature, 36.6 °C; pulse, 76 beats/min; respiratory rate, 18 breaths/min; and blood pressure, 128/82 mmHg. She appeared well-nourished and did not appear acutely ill. No palpable lymphadenopathy was detected in the cervical, axillary, or inguinal regions. Mucous membranes, conjunctivae, nails, and external genitalia were uninvolved. Dermatological examination revealed multiple erythematous patches of varying sizes on the neck and trunk. Some patches exhibited annular and gyrate configurations with mild scaling and well-defined borders, scattered with pinpoint pustules. The anterior chest showed large, dark erythematous plaques with crusting, scaling, and pinpoint pustules, but no vesicles or bullae were observed (Figure 1). Additional investigations were performed to differentiate inflammatory from autoimmune dermatoses.

**Figure 1:**
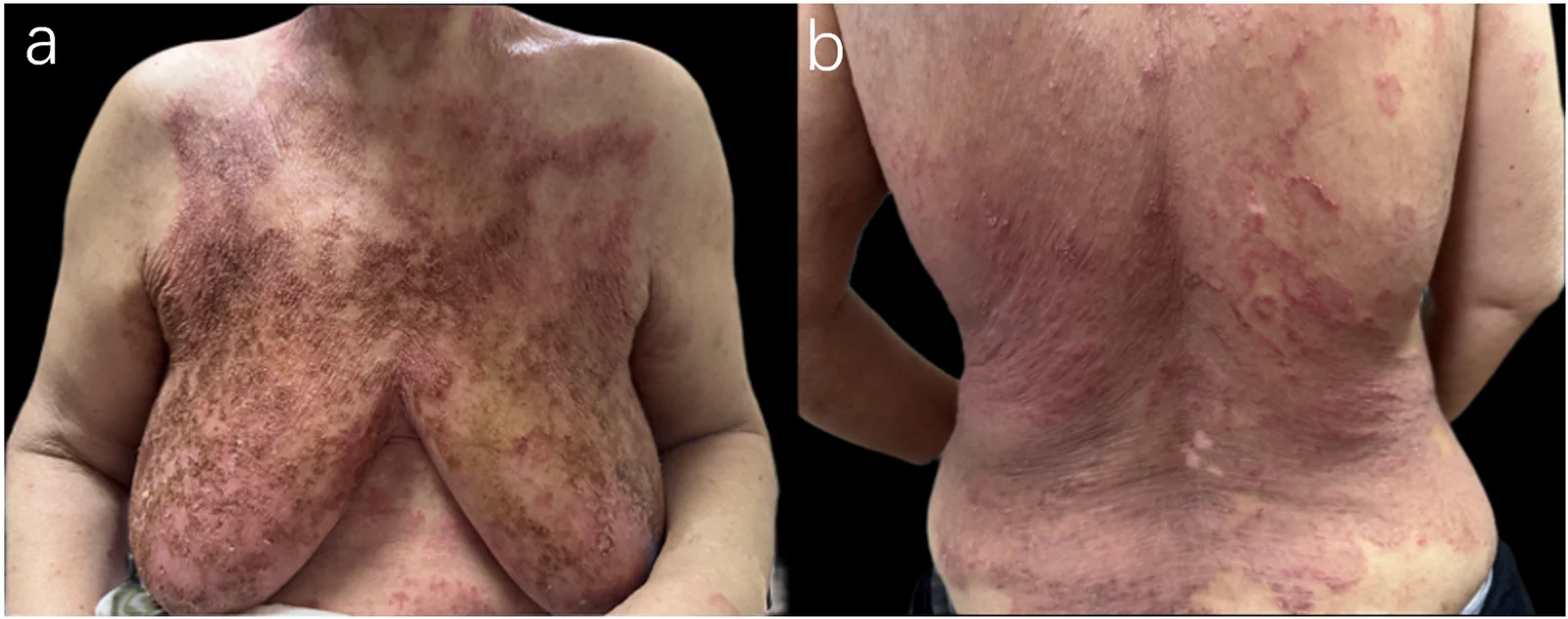
Clinical presentation of the patient during the initial consultation. **(a)** Multiple erythematous patches of varying sizes on the neck and trunk, some exhibiting annular and gyrate configurations with mild scaling and well-defined borders. **(b)** Anterior chest showing large, dark, erythematous plaques with crusting, scaling, and scattered pinpoint pustules. No vesicles or bullae were observed.

Routine laboratory findings, including complete blood count, C-reactive protein, procalcitonin, liver and renal function tests, and fasting blood glucose levels, were within normal limits. Serological screening for hepatitis B, syphilis, and human immunodeficiency virus (HIV) was negative. Chest radiography revealed no pulmonary infiltrates or nodules.

Histopathological examination of a skin biopsy from the right back revealed hyperkeratosis, acanthosis with elongation and fusion of rete ridges, and chronic inflammatory cell infiltration with neutrophils in the superficial dermis, accompanied by vascular dilation and congestion. Direct immunofluorescence (DIF) test results were negative for IgA, IgM, IgG, C3, and fibrinogen, effectively ruling out autoimmune bullous diseases.

Mycological examination of skin scrapings from the chest lesions revealed branched and septate hyphae under direct microscopy. Specimens inoculated onto Sabouraud dextrose agar (SDA) and incubated at 25 °C yielded a single pure fungal isolate. The colonies were subcircular, with a broad peripheral zone of white, floccose aerial hyphae; the central portion appeared grayish-brown, powdery, with radiating folds and scattered white aerial proliferative foci (Figure 2). Lactophenol cotton blue staining revealed septate hyphae with conidiogenous cells arising terminally or laterally from the hyphae, producing characteristic chains of smooth, globose conidia in a beadlike arrangement (Figure 3). mNGS was performed on superficial skin scrapings from active inflammatory lesions using the PMseq pathogen high-throughput gene detection platform. After sample reception, total nucleic acids were extracted. To optimize fungal cell wall lysis, enzymatic digestion combined with a bead-beating method was employed for DNA extraction. Following nucleic acid quality control, sequencing libraries were constructed and sequenced using a domestic high-throughput sequencing platform. Raw sequencing reads underwent quality trimming, low-quality sequence filtering, and the removal of human host sequences. The remaining non-host clean reads were taxonomically annotated using the PMseq bioinformatics pipeline against the Pathogen Metagenomics Database (PMDB), a clinical-grade pathogen reference database. A total of 78,672,713 high-quality clean reads were generated (Q30: 96.00%), and both the positive and negative controls passed quality validation. The theoretical detection sensitivity for fungi was 1.0E + 00 copies/mL (Supplementary Table S1). mNGS of lesional scrapings identified *M. trigonosporus* with 1,463 sequence reads and a relative abundance of 2.97% (Table 1). The 1,463 mapped reads were distributed across nearly the entire 32-Mbp reference genome of M. trigonosporus, with detectable signals in almost every 100-Kbp sliding window (4–14 reads per window) and an overall genome coverage of 0.3036% (Supplementary Figure S1). This multilocus dispersion pattern supports species-level assignment. Based on the integration of direct microscopy, culture, histopathology, and mNGS results, a definitive diagnosis of cutaneous infection by *M. trigonosporus* was established.

**Figure 2:**
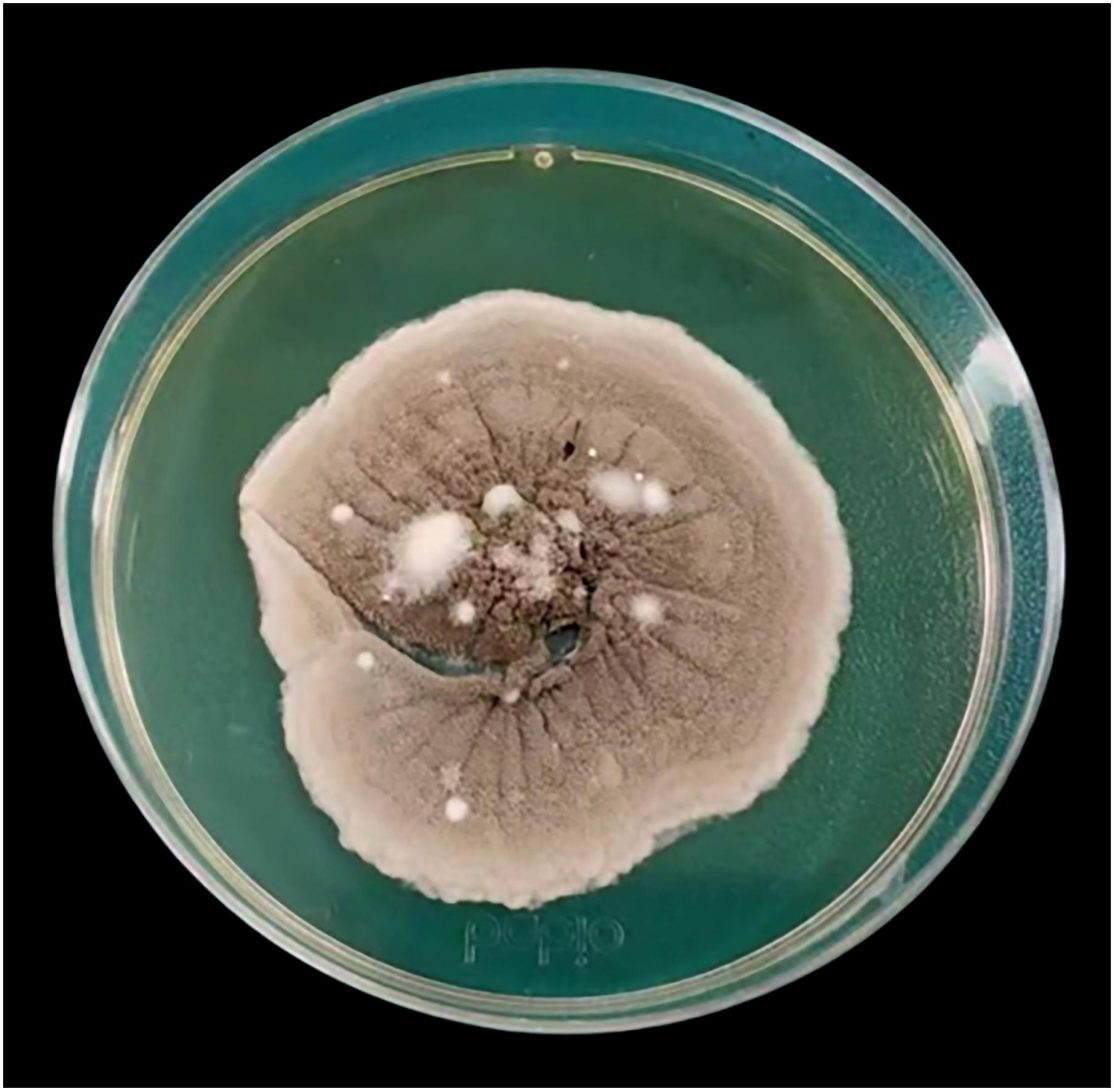
Colonial morphology of the isolated fungus on SDA incubated at 25 °C. Colonies were subcircular, with a broad peripheral zone of white, floccose aerial hyphae; the central portion appeared grayish-brown, powdery, with radiating folds and scattered white aerial proliferative foci (after 14 days of incubation).

**Figure 3:**
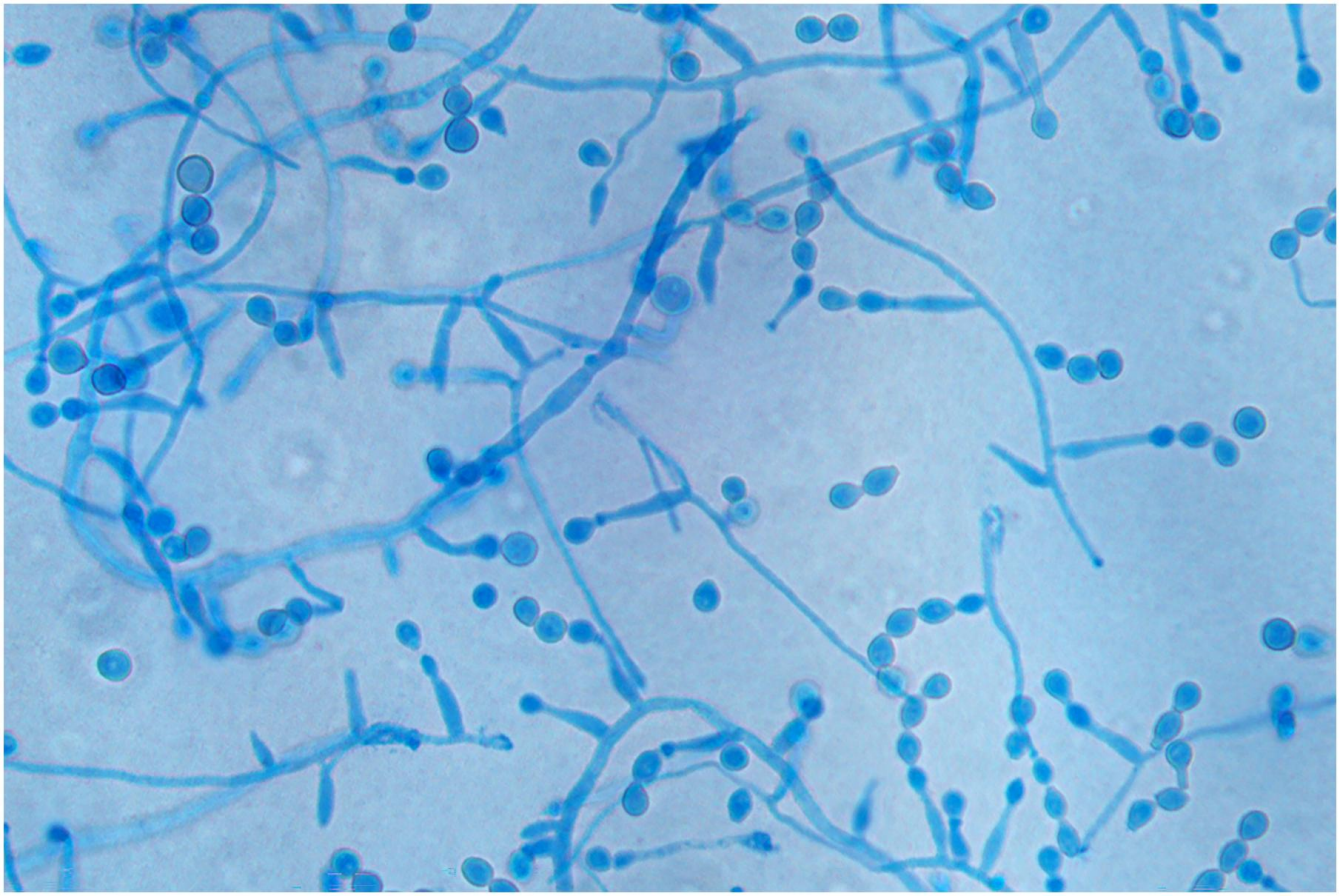
Microscopic morphology of the isolate (lactophenol cotton blue mount, ×400). Septate hyphae with conidiogenous cells arising terminally or laterally from hyphae, producing characteristic chains of smooth, globose conidia in a beadlike arrangement.

**Table 1:** mNGS results of the skin lesion specimen.

Genus	Species			
type	Reads	Pathogen	Reads	Coverage
*Microascus*	1,463	*M. trigonosporus*	1,463	2.97%

On the second day after admission, a fungal smear examination revealed septate hyphae. Based on these findings and the clinical presentation, a presumptive diagnosis of tinea corporis was made, and empirical oral fluconazole (50 mg once daily) was initiated, along with topical butenafine hydrochloride cream (1%) applied twice daily. After 2 weeks of treatment, although pruritus was partially alleviated, there was no significant improvement in the cutaneous lesions. Based on the findings of fungal culture and mNGS, *M. trigonosporus* was identified, prompting a modification of the antifungal regimen to oral itraconazole at a dosage of 200 mg once daily. Following 4 weeks of itraconazole therapy, the skin lesions showed marked regression, and pruritus was substantially relieved. Oral itraconazole was continued for an additional four weeks, resulting in complete resolution of the rash. No adverse events, including hepatotoxicity or visual disturbance, were reported during this period. Repeat fungal smears and cultures obtained from the previously affected chest area both yielded negative results. The patient reported full adherence to the treatment regimen and denied any adverse effects, including nausea, vomiting, visual disturbances, and photosensitivity. At the 1-year follow-up, no unexpected events were observed, and there was no evidence of recurrence of skin lesions (Figure 4). A concise clinical timeline is presented in Table 2.

**Figure 4:**
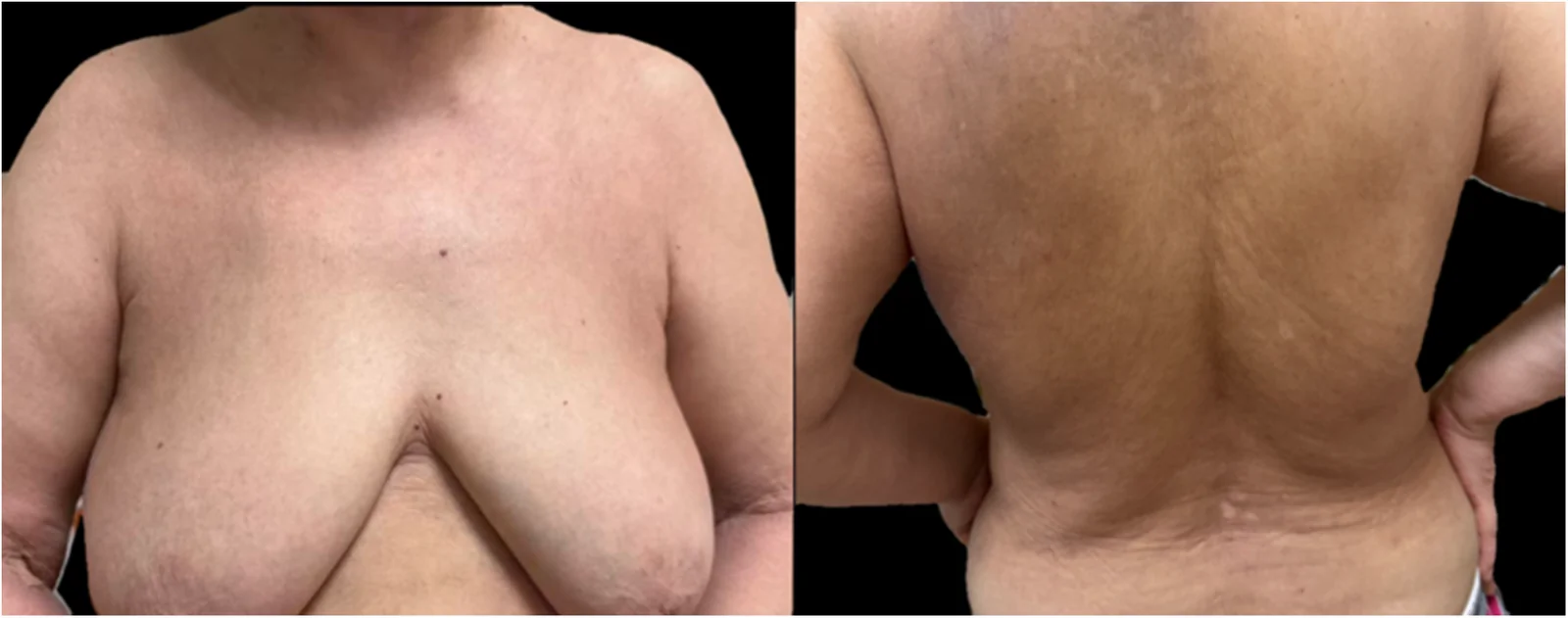
Follow-up clinical image of the patient one year after completion of itraconazole therapy. Complete resolution of the skin lesions was observed, with no evidence of recurrence.

**Table 2:** Clinical timeline of *M. trigonosporus* cutaneous infection.

Time point	Clinical events, examinations, and therapeutic interventions
2 months before initial presentation	The patient developed erythematous papules and plaques on the neck and trunk without any apparent predisposing factors. Prior to our evaluation, the patient had applied topical halometasone cream (0.05%) continuously for two months, with no improvement in the skin lesions
Day 1 of initial presentation	Relevant laboratory tests and examinations were performed; specimens were simultaneously collected for fungal smears, fungal cultures, mNGS, and skin biopsy
Day 2	The fungal smear was positive, revealing septate hyphae. In conjunction with the clinical manifestations, a presumptive diagnosis of tinea corporis was made, and empirical therapy was initiated with oral fluconazole (50 mg once daily) and topical butenafine hydrochloride cream (1%) twice daily
Day 5	Skin histopathology revealed hyperkeratosis, acanthosis, and chronic inflammatory cell infiltration in the dermis; direct immunofluorescence was negative, excluding autoimmune bullous diseases
Day 14	Both mNGS and fungal culture identified *M. trigonosporus*; the patient's skin lesions showed an unsatisfactory response to fluconazole therapy, and the regimen was switched to oral itraconazole 200 mg once daily (voriconazole was avoided because of the patient's history of glaucoma)
After 4 weeks of itraconazole therapy	Repeat liver function tests revealed no abnormalities; most skin lesions regressed, and pruritus was significantly relieved
After 8 weeks of itraconazole therapy	The skin lesions completely resolved, with no drug-related adverse events reported; itraconazole was subsequently discontinued
1 month after discontinuation	The skin lesions remained well resolved, with no new lesions or symptomatic complaints
1 year after discontinuation	Follow-up visits revealed no recurrence of skin lesions

## 3 Discussion

*M. trigonosporus* is a rare, emerging, opportunistic filamentous fungus. Previously reported invasive infections have occurred predominantly in solid organ transplant recipients, with pulmonary involvement as the main manifestation, while primary cutaneous infection is extremely rare (8). Consistent with the present case, members of the *Ochroconis–Microascus* complex have been reported to cause localized primary cutaneous infections in non-transplant, immunocompetent hosts, with favorable responses to standard systemic antifungal therapy (9, 10). This suggests that when the host immune function is not severely impaired, these fungi tend to present as localized skin infections, with systemic dissemination being exceptionally uncommon. We report a case of a localized primary cutaneous *M. trigonosporus* infection in a non-transplant, immunocompetent patient, expanding the known host range of this pathogen and demonstrating that such infections can occur in individuals without overt immunodeficiency.

Currently, two main pathogenic hypotheses exist for cutaneous *M. trigonosporus* infection: direct exogenous fungal inoculation and colonization through skin or mucosal microtrauma, and secondary cutaneous involvement following hematogenous dissemination from an occult pulmonary focus (4). The present patient had no respiratory symptoms or abnormal pulmonary imaging findings, which strongly supports the *in situ* inoculation mechanism.

This study had certain limitations in terms of clinical evaluation. In this case, quantitative immune function assays, including T lymphocyte subset analysis (CD4⁺ and CD8⁺ counts and their ratio) and serum immunoglobulin quantification (IgG, IgA, and IgM), were not performed. As the patient's immune status was assessed only by history and routine laboratory tests, subclinical immune deficits could not be completely excluded.

In this case, mNGS detected 1,463 reads of *M. trigonosporus*, but the relative abundance was only 2.97%, a relatively low value, raising the possibility of interference from cutaneous colonization or environmental contamination. However, the skin has a low microbial biomass, limiting the microbial DNA yield from scrapings or swabs (11, 12). Nonetheless, the combination of positive fungal culture, characteristic microscopic morphology, supportive histopathology, and a clear therapeutic response provided sufficient evidence to establish the diagnosis, mitigating the uncertainty associated with low mNGS read abundance. Owing to limited laboratory resources, the mNGS results were not confirmed by species-specific quantitative real-time polymerase chain reaction. In addition, independent phylogenetic analysis using tools such as Molecular Evolutionary Genetics Analysis (MEGA) was not performed. Nevertheless, the alignment of the PMseq pipeline against the PMDB reference database, combined with morphological identification, provided sufficient confidence for species-level assignment.

*Microascus* species generally exhibit intrinsic resistance to fluconazole; therefore, fluconazole is not recommended as an empirical therapy for infections caused by these fungi (3, 4, 13). Consistent with this resistance profile, the patient in this case showed no improvement in skin lesions after 2 weeks of standard fluconazole treatment, indicating that fluconazole was ineffective against the infection. Based on previous *in vitro* susceptibility data and clinical case reports, itraconazole has stable *in vitro* activity against some *Microascus* strains and has promising clinical application evidence and successful cases of localized cutaneous *Microascus* infections (1, 14). Voriconazole demonstrates superior *in vitro* activity against *M. trigonosporus* and is considered a first-line agent for invasive *Microascus* infections (15). However, owing to its well-documented ocular adverse effects, voriconazole is not suitable for patients with coexisting glaucoma. In this study, by integrating evidence-based susceptibility data, clinical efficacy, and individualized safety considerations, the treatment was switched to sequential itraconazole therapy, ultimately achieving complete resolution of the lesions without recurrence. This case confirms that itraconazole can be effectively used for localized, non-invasive cutaneous *M. trigonosporus* infection, and that voriconazole may serve as a salvage option for refractory cases. Given the substantial interstrain variability in antifungal susceptibility among rare fungal species, standardized susceptibility testing should be performed whenever possible to guide individualized precision therapy.

The cutaneous manifestations of rare filamentous fungal infections lack specificity; annular erythema, papules, pustules, and other presentations overlap considerably with those of various inflammatory dermatoses, which is a major reason for clinical misdiagnosis and missed diagnoses (16–18). Some atypical presentations of filamentous mycoses can mimic neoplastic skin lesions (19). In the absence of a clear etiology, the indiscriminate use of topical glucocorticoids may suppress local cutaneous immune defenses, leading to chronic, non-healing lesions and insidious disease progression. Traditional fungal culture has a long turnaround time, and species identification is highly dependent on the operator’s experience. In contrast, mNGS enables unbiased, high-throughput pathogen screening and can rapidly identify rare fungi that are difficult to detect using conventional methods, providing critical supplementary diagnostic value for challenging atypical cutaneous fungal infections.

In summary, for chronic annular or pustular skin lesions that do not respond to glucocorticoid therapy, clinicians should broaden the differential diagnosis and remain alert to the possibility of rare filamentous fungal infections. When *Microascus* species are clinically detected, the empirical use of fluconazole should be avoided. A multimodal diagnostic approach combining fungal culture, morphological observation, and mNGS can significantly improve the diagnostic yield of rare cutaneous mycoses. When formulating antifungal regimens, clinicians should carefully balance the antimicrobial activity of the agents against the individual patient safety profiles, thereby achieving standardized and personalized management of rare fungal diseases.

## 4 Patient perspective

Approximately 2 months ago, I noticed some red patches and small pus-filled bumps on my anterior chest. They did n't hurt, but were quite itchy. I went to a local hospital, and the doctor prescribed a cream (halometasone) to apply. I used it as instructed, but the rash did n't get better. Instead, it slowly spread to my neck and back, and new bumps continued to appear. I also tried some traditional Chinese medicine, but that did n't help either. I was very worried and could n't sleep well because of the itching.

When I came to this hospital, the doctors were very thorough in their examination. They took samples from my skin, performed several tests, and even used a new technology called mNGS to determine the exact cause of the infection. At first, I was prescribed oral fluconazole and a topical cream, but after 2 weeks, my skin showed minimal improvement. I felt slightly discouraged. Then, the doctors informed me that the test results revealed a rare fungus, and they switched my medication to itraconazole.

After approximately 4 weeks of taking itraconazole, I noticed that my skin was gradually clearing up, and the itching had reduced. By the eighth week, all the rashes had completely disappeared. I was so relieved and happy. During the entire treatment process, the doctors explained everything clearly, and the nurses were very kind and helpful. I did not experience any side effects from the medication, for which I was very grateful.

It has been a year since I finished the treatment, and the rash has never returned. I am very thankful to the medical team for their expertise and care they provided. I hope that by sharing my case, other patients with similar skin problems can receive the correct diagnosis and treatment sooner.

## Data Availability

The original contributions presented in this study are included in the article/Supplementary Material. Further inquiries can be directed to the corresponding author.
