# Supplementary material for: Case Report: Primary cutaneous infection caused by Microascus trigonosporus
Source: Front Med (Lausanne). 2026 Sep 24;13:1963973. doi: 10.3389/fmed.2026.1963973 (PMC13646135; doi:10.3389/fmed.2026.1963973)
Supplement: Supplementary file 1 [file Table1.docx]

| Supplementary Figure 1:Sequencing data quality control. | | | | | | | |
| --- | --- | --- | --- | --- | --- | --- | --- |
| Total sequencing reads | Microbial reads | Host removal | Human cell content (cells/mL) | Positive control | Negative control | Internal control detected | Q30 (%) |
| 78,672,713 | 85,971 | - | 1.9E+06 | pass | pass | Yes | 96.00 |
